# Fabrication and Properties of Electrospun Collagen Tubular Scaffold Crosslinked by Physical and Chemical Treatments

**DOI:** 10.3390/polym13050755

**Published:** 2021-02-28

**Authors:** Xuefei Chen, Jie Meng, Huaizhong Xu, Masaya Shinoda, Masanori Kishimoto, Shinichi Sakurai, Hideki Yamane

**Affiliations:** 1 Deptartment of Biobased Materials Science, Kyoto Institute of Technology, Kyoto 606-8585, Japan; d9861501@edu.kit.ac.jp (J.M.); shin@kit.ac.jp (S.S.); 2 Nitta Gelatin Inc., Osaka 581-0024, Japan; ma-shinoda@nitta-gelatin.co.jp (M.S.); ma-kishimoto@nitta-gelatin.co.jp (M.K.)

**Keywords:** electrospinning, collagen tube, ammonia treatment, dehydrothermal treatment, glutaraldehyde treatment, structure, denaturation, crosslink, properties

## Abstract

Tissue engineered scaffold was regarded as a promising approach instead of the autograft. In this study, small diameter electrospun collagen tubular scaffold with random continuous smooth nanofibers was successfully fabricated. However, the dissolution of collagen in concentrated aqueous (conc. aq.) acetic acid caused to the serious denaturation of collagen. A novel method ammonia treatment here was adopted which recovered the collagen triple helix structure according to the analysis of IR spectra. Further dehydrothermal (DHT) and glutaraldehyde (GTA) treatments were applied to introduce the crosslinks to improve the properties of collagen tube. The nanofibrous structure of collagen tube in a wet state was preserved by the crosslinking treatments. Swelling ratio and weight loss decreased by at least two times compared to those of the untreated collagen tube. Moreover, tensile strength was significantly enhanced by DHT treatment (about 0.0076 cN/dTex) and by GTA treatment (about 0.075 cN/dTex). In addition, the surface of crosslinked collagen tube kept the hydrophilic property. These results suggest that DHT and GTA treatments can be utilized to improve the properties of electrospun collagen tube which could become a suitable candidate for tissue engineered scaffold.

## 1. Introduction

Nervous and vascular diseases are the prevalent diseases worldwide which cause the functional disability of the patients and death [1,2]. In these cases, the clinical gold standard for the treatment of the injured nerves and diseased vessels is the autograft, which can give its ready availability, microenvironment and native growth factors [3,4]. Nevertheless, there can often be associated with sacrificing function at the donor site or lack of suitable donor tissue. For these reasons, tissue engineered tubular scaffold, which offsets the inherent disadvantage, has been proposed to be a promising approach to autograft repairs, particularly in short defects [5,6]. 

Artificial tubular scaffold bridges the damaged tissue ends that contribute to the tissue regeneration. An ideal scaffold should be fabricated into a desired diameter, possess biocompatibility, permeability and flexibility and have a customizable biodegradable matrix [2,7]. The main challenge is that scaffold can mimic the components and structures of extracellular matrices (ECM), which markedly affects the cell responses and the diffusion of nutrients [8,9].

Native ECM is the nanofibrous porous matrix composed of the protein and polysaccharide [10]. The most promising method of fabricating the structure to mimic ECM is electrospinning. Electrospinning is a simple and efficient method to produce continuous nanofibers [11]. During electrospinning, a high voltage is applied to a polymer solution or melt to eject it as an electrically charged fluid jet, which travels toward a grounded collector, solidifies in fibrous form with solvent evaporation or cooling [12,13]. Electrospun nanofibers with high porosity, high surface area to volume ratio, allowing cell adhesion and the efficient transportation of nutrients and oxygen have been widely used in the tissue engineering, drug delivery, biosensing, antibacterial applications and so on [14,15,16,17,18,19,20]. In addition, in the fight against the Coronavirus disease 2019 (COVID-19), electrospun nanofibers attract much more attention in the self-disinfecting personal protective equipment (PPE), such as face masks [21,22]. On the other hand, characterizations of electrospun scaffolds can be tailored in accordance with the purpose of their use by adjusting the materials, processing parameters, environmental conditions [12,23].

Even though varied synthetic materials such as polycaprolactone, polylactic acid, have been fabricated into tissue scaffolds by electrospinning, the major obstacle in usage of synthetic materials lies in their biocompatibility [24,25,26]. Based on this fact, the collagen, a primary component of native ECM, can provide a supportive environment analogous to natural ECM. In addition, with excellent biocompatibility, biodegradability and hydrophilicity, collagen has attracted great attention in tissue engineering [9,27]. Collagen characteristic conformation is triple helix structure consisting of three polypeptide chains intertwined in a right-handed form, which gives the structure stability [28]. Generally, fluoroalcohols and acid solutions are used to dissolve collagen to meet the necessary viscoelastic response for electrospinning, which mostly result in denaturation of collagen [29,30]. Zeugolis et al. reported that 99% of collagen triple helix structure lost after electrospinning [31]. Electrospun collagen and collagen-based scaffolds show poor mechanical property, rapid degradation and higher water absorption.

Crosslinking treatment is a necessary step before electrospun collagen scaffolds can be utilized for tissue regeneration. Chemical crosslinking treatment glutaraldehyde (GTA) forms the crosslinks by the reaction of the free amine groups with the aldehyde groups of GTA [32,33]. It is easily available, effective, inexpensive and widely used to improve the stability of collagen scaffolds. Dehydrothermal (DHT) treatment, one of common physical treatments, introduces covalent bond between the collagen molecules by condensation reactions under high temperature [34]. Different crosslinking methods affect the characteristics regarding mechanical properties, degradation, etc., owing to the different mechanism, which should be considered depending on the applications [35,36].

The purpose of this study is to fabricate the suitable collagen tube for tissue engineering by electrospinning. Acetic acid, as the solvent, was used to dissolve collagen for avoiding toxicity. The optimal parameters of electrospinning were obtained based on the fiber morphology. After fabrication of the collagen tube, a new method ammonia treatment was adopted to neutralize the residual acetic acid and IR spectra was used to analyze the effect. Various crosslinking treatments (GTA and DHT) were applied to introduce the crosslinks to enhance the properties of collagen tube. The effects of various treatments on the structure and properties of collagen tubes were investigated, including fiber morphology, chemical structure, mechanical properties, swelling ratio, degradation and hydrophilicity.

## 2. Experiments

### 2.1. Materials

The collagen gel was from the pork tendon extracted by pepsin. It was freeze dried using a lyophilizer (EYELA, FDU-2110) and stored in a vacuum glass desiccator at a controlled temperature 20 ± 2 °C before use.

Aqueous ammonia solution (28%, 14.9 M), aqueous glutaraldehyde solution (25% in water) and acetic acid (99.0 wt%) were purchased from NACALAI TESQUE, INC. (Kyoto, Japan).

### 2.2. Fabrication of Electrospun Collagen Tube

The 12 wt% of collagen solution was prepared by dissolving lyophilized collagen in a solvent mixture of acetic acid and water (volume ratio, 2:3) and stirring for 24 h at room temperature. This solution was then transferred into a 1 mL plastic syringe with a 22-gauge metal needle. The optimal process parameters (voltage, flow rate and needle-collector distance) were determined based on the fiber morphology and stability of the electrostatic jet during electrospinning. A positive voltage of 15 KV was applied to the needle using a high voltage power supply (Model-600F, Pulse Electronic Engineering Co., Ltd., Noda, Japan). Collagen solution was extruded through the needle using a syringe pump at a flow rate of 3 μL/min. A stainless cylindrical steel mandrel (diameter: 2 mm, length: 10 cm) as a collector was placed at a distance of 12 cm from the needle tip. The rotation rate of the mandrel was set at 300 rpm. A grounded aluminum sheet was placed at the back of mandrel to induce the fiber collection on the mandrel. The spinning time is 35 min for every tube sample under a controlled atmosphere 20 ± 2 °C and 35 ± 2% RH.

After fabrication of the collagen tubes, three kinds of treatments were applied to the tubes, “ammonia treatment”, “dehydrothermal treatment” and “glutaraldehyde treatment”.

### 2.3. Ammonia Treatment

Ammonia treatment was carried out by placing the collagen tubes in a sealed glass desiccator which contained 100 mL of aqueous ammonia solution. The collagen tubes on the mandrel were placed on a holed ceramic shelf and exposed in the ammonia vapor for 60 min at room temperature. After being exposed, the collagen tubes were placed in a vacuum oven (Yamato ADP 200, Tokyo, Japan) (0.5 KPa) for 6 h to remove residual ammonia before various measurements. The optimal ammonia treatment time was determined to be 60 min comparing with other series of conditions (20 min, 120 min, 240 min or 480 min) according to the tensile strength (Figure S1).

### 2.4. Ammonia-Dehydrothermal (DHT) Treatment

Ammonia treatment was applied to the collagen tubes in advance and then dehydrothermal (DHT) treatment was adopted. The collagen tubes on the stainless steel mandrel were exposed to ammonia vapor for 60 min at room temperature. After ammonia treatment, collagen tubes were kept in an oven (Yamato ADP 200, Japan) under vacuum (0.5 KPa) for 24 h at room temperature and slowly heated to 125 °C and kept for 3 days. Then, the oven was cooled down slowly to room temperature under vacuum state. This condition of treatment showed higher crosslinking efficiency compared with other series of conditions (105 °C or 145 °C) (Figure S2).

### 2.5. Ammonia-Glutaraldehyde (GTA) Treatment

Ammonia treatment was applied to the collagen tubes in advance and glutaraldehyde (GTA) treatment was further applied. The collagen tubes on the mandrel were exposed to ammonia vapor for 60 min and then placed on a holed ceramic shelf in a sealed glass desiccator containing 100 mL of GTA solution. The collagen tubes were exposed in GTA vapor at room temperature for 3 days. This condition of GTA treatment was confirmed to give better performance compared with other series of conditions (1 day, 3 days or 4 days) (Figure S3). After treatment, the collagen tubes were placed in a vacuum oven (Yamato ADP 200, Japan) (0.5 KPa) for 6 h to remove all residual ammonia and GTA before various measurements.

**Figure 1:**
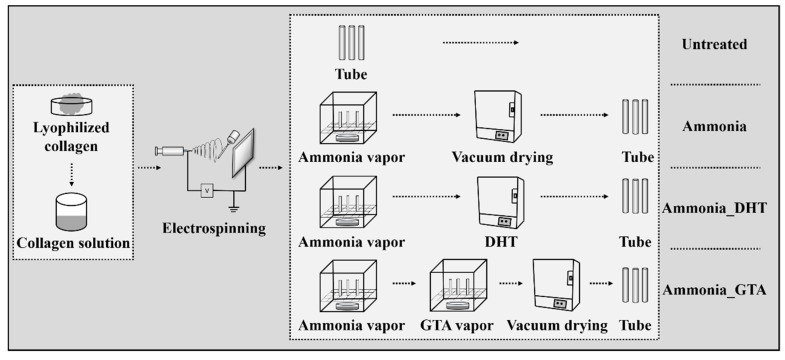
A schematic image of the preparation of collagen tube and various treatments were applied.

Figure 1 shows the schematic image of the preparation of collagen tubes with various treatments. The treated collagen tubes were stored in a glass desiccator with a controlled atmosphere 20 ± 2 °C and 50 ± 2% RH at least 48 h before analysis.

### 2.6. Characterizations

#### 2.6.1. Morphological Observations

Morphological observations were carried out by using a scanning electron microscope (SEM, KEYENCE, Osaka, Japan, VE-7800) at an accelerated voltage of 10 kV. The tubes were coated with an ultra-thin layer of gold (IB-2, Eiko Engineering, Co. Ltd. Yamazaki, Japan) prior to the observation. The average diameter was determined by using an Image analysis software (ImageJ, National Institutes of Health, Bethesda, MD, USA). More than 100 random fibers from the captured images were selected to analyzing the diameter. The diameter values are reported as means ± standard deviations.

#### 2.6.2. Mechanical Properties

**Figure 2:**
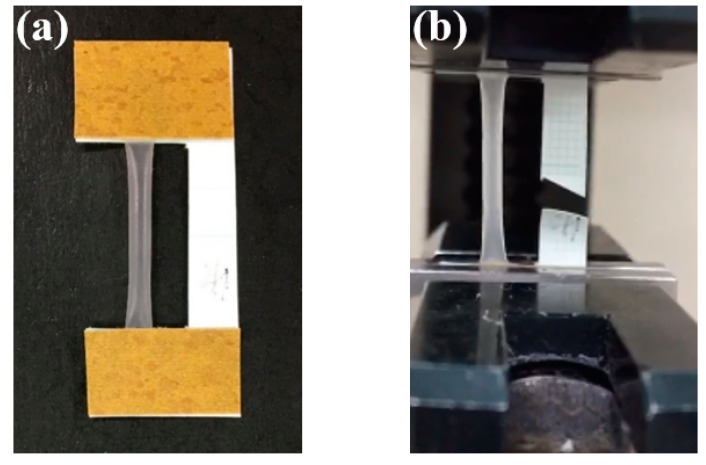
The collagen tube fixed on the paper frame (**a**) and that clamped by the tensile tester (**b**).

Mechanical properties of the collagen tubes were evaluated in a wet state using a tensile tester (STA-1150, ORIENTEC, Tokyo, Japan). The collagen tube on the stainless steel mandrel was cut 3 cm in length and soaked in the deionized (DI) water for 15 min. The collagen tubes 3 cm in length were removed from the mandrel and immersed in DI water at 37 °C for 24 h to prepare the tensile test sample. The water on the surface was wiped with a filter paper just before tensile testing. The collagen tube was fixed on the paper frame and clamped by the tensile tester as shown in Figure 2. The testing-related parameters including thickness, weight and length of the tube were measured using a digital micrometer, a weighing scale and a ruler. The tube with a 20 mm initial clamping distance was stretched at a rate of 10 mm/min at room temperature. The stress–strain curve was given directly from the tester. The tensile strength, modulus and the elongation to break were determined from the stress–strain curves. The measurements were repeated five times for each sample from more than 3 mandrels.

#### 2.6.3. Swelling Ratio and Weight Loss

The 5 mm × 2 cm square pieces were cut from the tube. Swelling ratio and weight loss of the pieces were measured gravimetrically after immersing them in DI water at 37 °C for 24 h. The wet weight of sample was measured immediately after wiping the water on the surface with a filter paper. The sample was air-dried at the same condition for 24 h to get the weight in the dry state. The measurements were repeated five times for each sample from more than 3 mandrels. The swelling ratio and weight loss are defined as

(1) $$Swelling\;ratio=\frac{M_{w}-M_{d}}{M_{d}}$$

(2) $$Weight\;loss\;\left(\%\right)=\frac{M_{i}-M_{d}}{M_{i}}\times 100$$

where *M*_i_, *M*_w_ and *M*_d_ are the weights of sample before soaking in water, wet samples and sample dried after immersing in water.

#### 2.6.4. Fourier Transform Infrared Spectroscopy (FTIR)

Chemical structure of the electrospun collagen tube was analyzed by using a Fourier transform infrared spectroscopy (FT/IR-4700 TKG, JASCO, Tokyo, Japan) in an attenuated total reflection (ATR) mode with the Ge crystal plate. The spectra were recorded from 4000 to 750 cm^−1^ with an average of 32 scans and a resolution of 4 cm^−1^. The measurements were repeated three times for each sample from more than 3 mandrels.

#### 2.6.5. Water Contact Angle

The collagen tubes on the stainless steel mandrel were fixed on glass slides using the double side tape. The water contact angle was measured by the sessile drop method using SEO Phoenix 300 Touch Automatic Contact Angle Analyzer (KROMTEK, Suwon City, Korea). A volume of 1 µL distilled water was deposited on the sample surface and drops were video recorded. The contact angle was calculated according to the images at the time of 3 s. The measurements were repeated three times for each sample from more than 3 mandrels.

#### 2.6.6. Statistical Analysis

The results are presented as means ± standard errors. Statistical significance was tested using One Way ANOVA. Statistical significance was accepted at *p* < 0.05 and indicated in the figures as * *p* < 0.05.

## 3. Results and Discussion

### 3.1. FTIR Spectroscopy Analysis

#### 3.1.1. FTIR Spectroscopy Analysis of the Collagen Tubes as Electrospun

The viscoelastic response of the solution was critical for the smooth electrospun fibers. Native collagen solution behaves as gel because of the intermolecular association. To meet the necessary entanglement properties, fluoroalcohols and acid solutions have been used to dissolve collagen by sacrificing the collagen triple helix structure [37,38].

**Figure 3:**
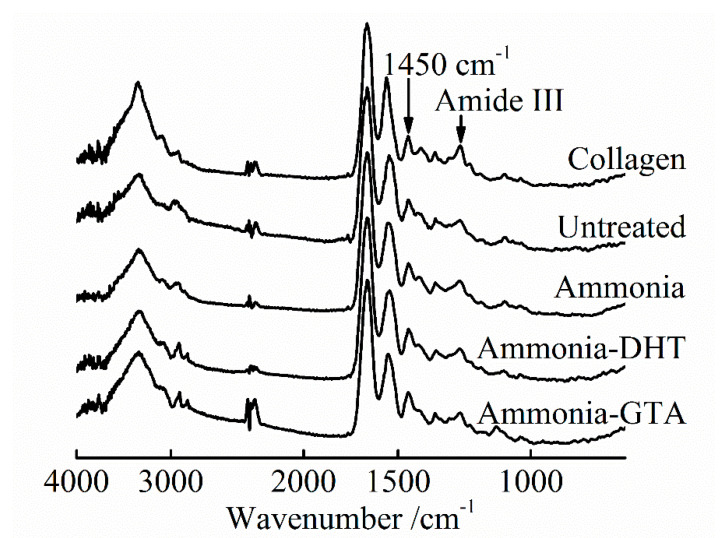
FTIR-ATR spectra of original lyophilized collagen and electrospun collagen tubes before and after various treatments.

**Figure 4:**
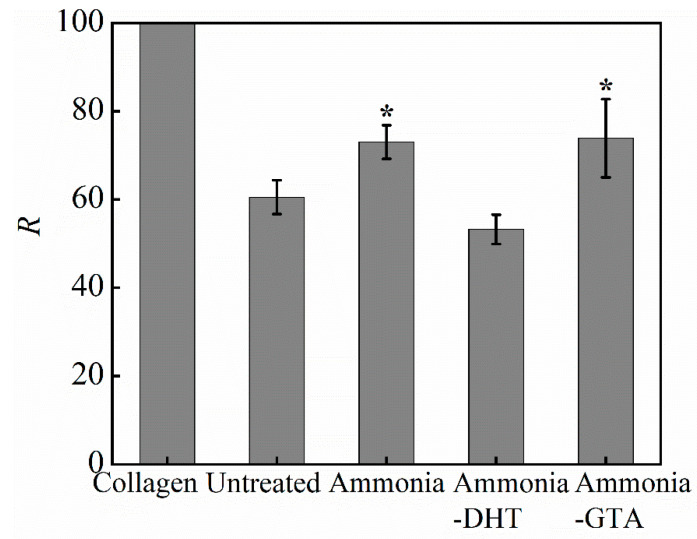
The ratio *R* representing the degree of denaturation of original lyophilized collagen and electrospun collagen tubes before and after various treatments. * *p* < 0.05, collagen tubes with ammonia treatment and ammonia-GTA treatment are significantly different from untreated collagen tube.

Lyophilized pork collagen was dissolved in concentrated aqueous (conc. aq.) acetic acid in this study. Detailed change of the chemical structure of collagen after electrospinning was studied by FTIR-ATR spectra in Figure 3 and Figure 4. Characteristic bands of collagen IR spectra include amide I band (~1650 cm^−1^), amide II band (~1550 cm^−1^) and amide III band (~1240 cm^−1^), which are indicative of change of collagen structure [39,40]. The amide III band is sensitive to the change of collagen triple helix structure which is assigned to the C-N stretching and N-H in-plane deformation vibration [35,41]. It can be seen that the intensity of amide III band of original lyophilized collagen was much higher than that of electrospun collagen tube shown in Figure 3. It indicated that the triple helix structure was damaged by dissolving collagen in conc. aq. acetic acid.

For directly descripting the degree of collagen denaturation, the ratio *R* of amide III peak area to 1450 cm^−1^ peak area was calculated.

(3) $$R=A_{amide\;III}/A_{1450}$$

The intensity of the peak at ~1450 cm^−1^ which corresponds to the variation of C-H including CH_2_ deformation and CH_3_ asymmetric deformation was kept unchanged. The intensity of amide III decreased with the increase of denaturation as described above. Based on this, the ratio *R* decreased with increasing the degree of denaturation [42]. Figure 4 descripts the ratio of two peaks area after assigning the *R* value of original lyophilized collagen as 100%. It can be seen that the peak area of amide III band was reduced to half, indicating that part of collagen lost triple helix structure after electrospinning. Dissolving collagen in conc. aq. acetic acid caused to the serious denaturation of collagen.

#### 3.1.2. FTIR Spectroscopy Analysis of the Collagen Tubes after Treatments

Ammonia treatment was adopted to neutralize the residual acetic acid which could be remained in the collagen fibers and obstruct the recovery of collagen triple helix structure. An electrically charged fluid jet from the tip of Taylor cone flies toward to the grounded target during the electrospinning process. In the meanwhile, the solvent evaporates and the fibers are formed [23]. However, because of the high boiling point of acetic acid 117.9 °C, acetic acid may not be completely evaporated [43]. From the IR spectra shown in Figure 3, the intensity of amide III band increased to some degree after ammonia treatment. The ratio *R* of ammonia treated collagen tube was higher than that of untreated collagen tube as shown in Figure 4. It confirmed that ammonia treatment facilitated the recovery of collagen triple helix structure by neutralizing the residual acetic acid. Ammonia treatment is one of the treatments necessary to reform the triple helix structure of the collagen.

Physical and chemical crosslinking treatments were applied after the application of ammonia treatment. DHT treatment is widely used to improve the properties of collagen-based materials because of its non-cytotoxicity [35,44]. It forms covalent bonds between the collagen peptide chains by condensation reactions under high temperature [34,45]. Compared with the ratio *R* of ammonia treated collagen tube, ammonia-DHT treated collagen tube had much lower ratio *R*. It showed that ammonia-DHT treatment aggravated the denaturation due to the high treatment temperature [34]. GTA treatment is a typically chemical crosslinking method which produces the crosslinks through the reaction of the free amine groups of lysine or hydroxylysine amino acids residues of collagen peptide chains with the aldehyde groups of GTA [33,46]. Because the reaction mechanism does not give any effect on the triple helix structure, the collagen tube with ammonia-GTA treatment showed a higher ratio *R* than that of ammonia-DHT treated collagen tube.

### 3.2. Morphological Analyses

#### 3.2.1. Morphology of the Collagen Tubes as Electrospun

**Figure 5:**
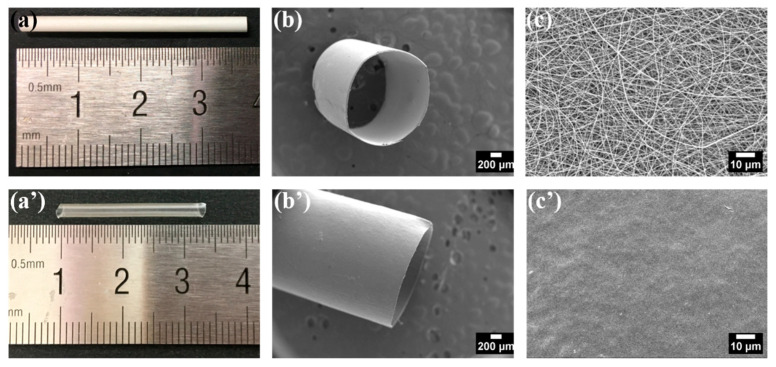
The gross appearance and SEM images of electrospun collagen tube before (previous row) and after (next row) immersing in water for 15 min. The gross appearance (**a**,**a’**), SEM images of collagen tube: entire (**b**,**b’**) and surface (**c**,**c’**).

The electrospun collagen tubular scaffold consisting of continuous collagen nanofibers has high porosity and high surface area to volume ratio, which can mimic the structure and component of native ECM. For achieving the optimum morphological structure, electrospinning parameters were determined according to the morphology of collagen nanofibers. Small diameter collagen tubes with 2 mm in inner diameter and 20 μm in thickness were successfully fabricated. Representative gross appearance and SEM images of electrospun collagen tube are shown in Figure 5a–c. Collagen tube consists of random oriented smooth nanofibers and interconnected pores. The fiber diameter was about 336 nm in average.

The electrospun collagen tubular scaffolds are prepared to use in the human body. Figure 5a’–c’ give the morphology of the electrospun collagen tube after immersing in water. The collagen nanofibers showed immediate swelling, collapsing and weak resistance against dissolution. The nanofibrous structure was destroyed and the surface of tube turned to be smooth. The serious denaturation of collagen during dissolution process in conc. aq. acetic acid was considered to be responsible for it. Almost half the collagen lost the triple helix structure after electrospinning from the FTIR spectra results. The recovery of the collagen triple helix structure and introduction of crosslinks could be a critical step to improve the properties required for tissue engineered scaffolds, such as morphological stability, degradability and mechanical property.

#### 3.2.2. Morphology of the Collagen Tubes after Treatments

**Figure 6:**
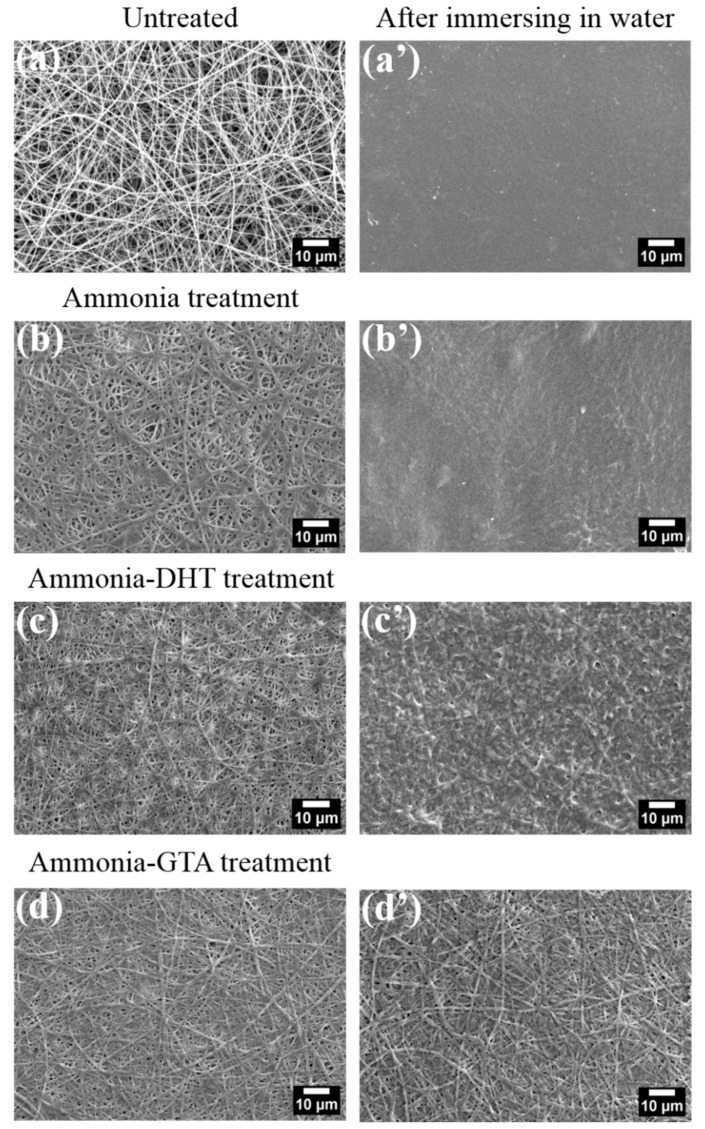
SEM images of electrospun collagen nanofibers before (left column) and after (right column) immersing in water for 1 day at 37 °C. Untreated (**a,a’**), ammonia treatment (**b**,**b’**), ammonia-DHT treatment (**c**,**c’**) and ammonia-GTA treatment (**d**,**d’**).

Due to the sensitivity to water contact, the treatments were performed by exposing electrospun collagen tubes in the vapor. Figure 6 shows the morphology of collagen fibers with various treatments in different states.

Electrospun collagen fibers were smooth and uniform as shown in Figure 6a. After ammonia treatment, collagen fibers became swollen and tended to be fused and stick together in Figure 6b. The fiber diameter increased from 336 ± 149 nm to 452 ± 149 nm as seen in Table 1. The reason for the phenomenon was that the water moisture contained in ammonia vapor was absorbed in the collagen fibers [47]. When the collagen tube was immersed in water for 1 day at 37 °C, the nanofibrous structure was destroyed in Figure 6b’. Surface of collagen tube became smooth and high porosity disappeared. There was no difference between the morphology of ammonia treated collagen tube and untreated collagen tube in a wet state. It meant that ammonia treatment did not improve the resistance against water, although IR analysis indicated that ammonia treatment facilitated the recovery of the collagen triple helix structure to some extent. Hence, additional crosslinking treatments also should be performed after ammonia treatment.

Figure 6c gives the morphology of collagen fibers after applying ammonia-DHT treatment. Fibers were thicker and tended to be fused compared to the untreated collagen fibers because of the influence of ammonia treatment as described above. After immersing in water, fibers tended to be fused together, although part of nanofibrous structures were persevered as shown Figure 6c’. It indicated that DHT treatment which introduced the limited crosslinks had an inconspicuous effect on protecting the nanofibrous structure in a wet state.

The morphology of collagen fibers with ammonia-GTA treatment is in Figure 6d. As expected, the fibers became flatter and fatter due to the co-existence of water moisture with ammonia vapor and GTA vapor during treatments. The detailed change of fiber diameter is in Table 1. After immersing in water for 1 day at 37 °C, it is noteworthy that the nanofibrous structure were preserved integrally in Figure 6d’. Furthermore, the fibrous form after 1 day soaking was similar to that before soaking. It demonstrated that ammonia-GTA treatment was an efficiency method to form crosslinks which preserved the collagen fibers from the damage of water.

### 3.3. Swelling Ratio and Weight Loss

**Figure 7:**
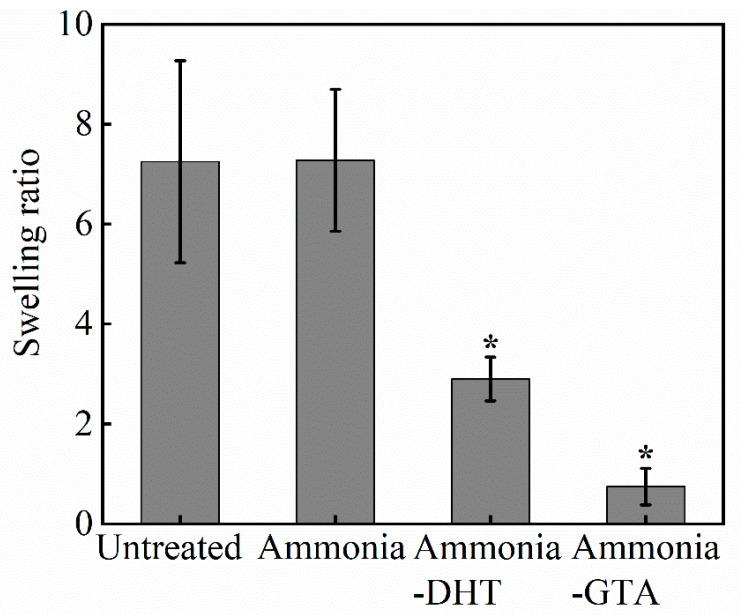
Swelling ratio of collagen tubes before and after various treatments. * *p* < 0.05, crosslinked collagen tubes are significantly different from untreated collagen tube.

The swelling ratios of electrospun collagen tubes before and after various treatments are shown in Figure 7. Untreated collagen tube showed an extremely high swelling ratio. Even ammonia treatment did not reduce it. The swelling ratio generally reflects the degree of crosslinking [48]. It indicated that collagen tube as electrospun did not have enough crosslinks to resist to water invasion. Moreover, ammonia treatment did not promote the formation of crosslinking but only the recovery of collagen triple helix structure. It is consistent with the poor morphological stability of ammonia treated collagen tube. In contrast, swelling ratios of collagen tubes with crosslinking treatments decreased significantly. The formation of crosslinks contributed to build a water-insoluble network which suppressed the swelling. A special focus was given on the swelling ratio of collagen tube with ammonia-DHT treatment. Because of the limited crosslinks introduced by DHT treatment, the collagen tube absorbed twice as much water as its dry weight, which gave rise to the damage of collagen fiber structure shown in Figure 6c’.

**Figure 8:**
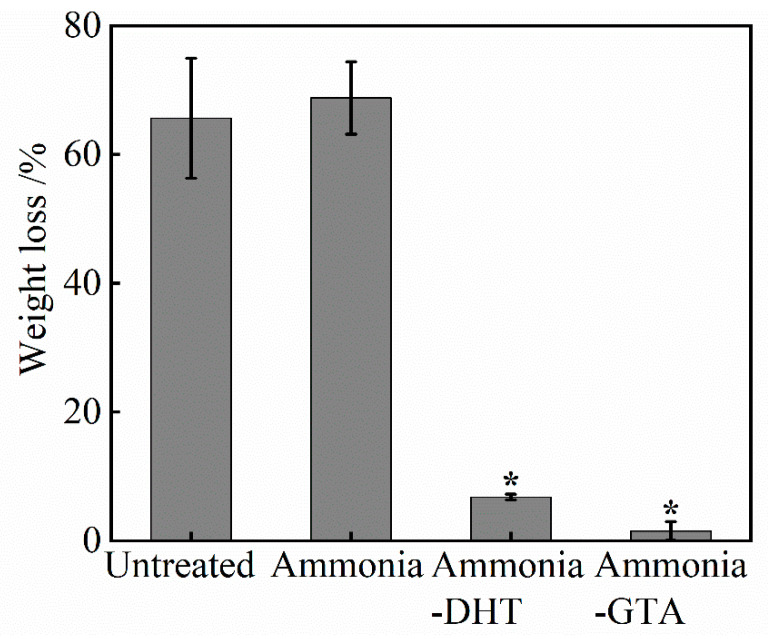
Weight loss of collagen tubes before and after various treatments. * *p* < 0.05, crosslinked collagen tubes are significantly different from untreated collagen tube.

The weight loss of collagen tubes before and after various treatments showed a similar trend in Figure 8. Nearly 70% of weight of untreated or ammonia treated collagen tube lost due to the dissolution of collagen fibers in water. Collagen tubes after crosslinking treatments revealed an obvious lower weight loss. In particular, a slight weight loss about 1% was found after ammonia-GTA treatment. By contrast, ammonia-DHT treated collagen tube had a higher weight loss about 7% due to the lower crosslinking efficiency. The above analysis demonstrated that DHT and GTA treatments can improve the water resistance of collagen tube by introduction of crosslinks. Swelling ratio and weight loss decreased by at least two times compared to those of untreated collagen tube.

### 3.4. Mechanical Properties

**Figure 9:**
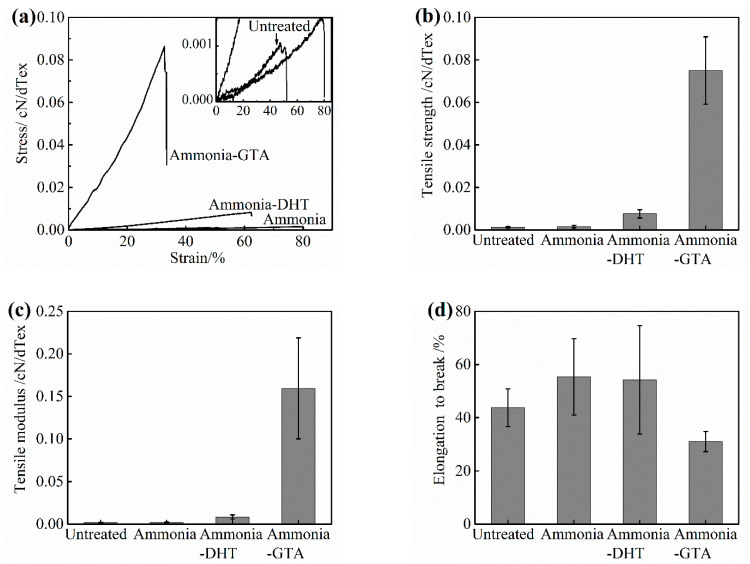
Mechanical properties of collagen tubes before and after various treatments in a wet state: (**a**) stress-strain curves, (**b**) tensile strength, (**c**) tensile modulus and (**d**) elongation to break.

Mechanical properties are critical physical performances which should be adequate for withstanding any type of stress under physiologic conditions [49]. The typical stress-strain curves of electrospun collagen tubes in a wet state before and after various treatments are plotted in Figure 9a. The curve shape of untreated collagen tube showed a flat stress grow-up with increasing strain. The tensile behavior reflected the weaker and softer properties of untreated collagen tube. After ammonia treatment, the curve shape was similar to that of untreated collagen tube. However, the collagen tubes with ammonia-DHT treatment or ammonia-GTA treatment showed much steeper stress growing. The tensile strength, tensile modulus and elongation to break are determined from the stress-strain curves and plotted in Figure 9b–d, respectively.

The untreated collagen tube showed lower tensile strength and tensile modulus because of the serious denaturation of collagen. After the application of ammonia treatment, the tensile strength and modulus kept unchanged. The reason could be that mechanical properties are associated with the crosslinks between collagen peptide chains. The denaturation of collagen disturbed the original molecular structure and broke down the regular interactions between peptide chains [50]. Although the ammonia treatment facilitated the recovery of collagen triple helix structure, it did not introduce enough crosslinks to improve the mechanical properties.

After crosslinking treatments, the tensile strength and modulus of collagen tubes were improved noticeably. The tensile strength of a native artery is reported to be about 1.50 MPa [49]. Tensile strength of collagen tubes with ammonia-DHT treatment and ammonia-GTA treatment were about 0.0076 cN/dTex (equal to 1.23 MPa) and 0.075 cN/dTex (equal to 5.25 MPa), respectively. The application of ammonia-GTA treatment made the tensile strength and modulus more than 10 times higher than those of untreated collagen tube. Regarding elongation to break, ammonia- GTA treatment decreased it because the crosslinks restricted the mobility of the peptide chains. The different effects brough by ammonia -DHT treatment and ammonia-GTA treatment were relative to the different crosslinking mechanism.

### 3.5. Water Contact Angle

**Figure 10:**
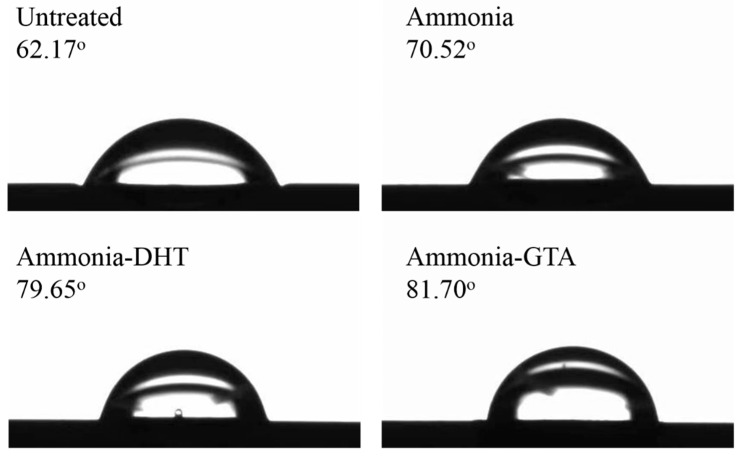
Water contact angle of collagen tubes before and after various treatments.

Surface property of tissue engineered scaffold is important for the cell response. To explore the effect of various treatments on the hydrophilic property of collagen tube surface, water contact angle was analyzed in Figure 10. The water contact angle of untreated collagen tube was about 62°, indicating that untreated collagen tube surface had a good hydrophilicity attributed to numbers of hydrophilic groups in the collagen structure such as amino group. Note that the water contact angle of untreated electrospun collagen tube was about 30° lower than that of pure collagen film in the literature [42], indicating the exposure of hydrophilic groups due to the damage of collagen structure after electrospinning [51]. Compared with the value of collagen tube as electrospun, the water contact angles increased after crosslinking treatment either by ammonia-DHT or ammonia-GTA treatment. The value increase could be associated with a decrease of hydrophilic groups in the collagen. Because DHT and GTA treatments introduce the crosslinks by the reactions in the free hydrophilic groups [33,52]. Although the water contact angles after various treatments increased, they were lower than 90°. It meant that the surface of collagen tube still showed the hydrophilic property.

As a suitable tissue engineered scaffold, its structure and properties must adapt to the real clinical applications. The above analysis has presented the positive effects of crosslinking treatments on the structure, degradability and mechanical properties of electrospun collagen tubes. Moreover, the scaffold needs to have good biocompatibility to support cell growth. Many studies had reported that GTA and DHT treatments had no obvious negative impact on the cell viability and supported cell adhesion, proliferation and spreading [44,53,54,55,56]. However, the slight cytotoxicity could be brought if the existence of residual GTA. Campiglio et al. confirmed that GTA treatment without proper preconditioning had the lower cell viability compared to 1-ethyl-3-(3-dimethylaminopropyl) carbodiimide/ N-hydroxysuccinimide (EDC/NHS) treatment [57]. Drexler et al. indicated that DHT treatment also had a lower cell number compared to EDC treatment because DHT treatment reduced the wetting ability [58]. Therefore, for a more comprehensive evaluation, future studies will be focused on the biocompatibility of eletrospun collagen tubes crosslinked by GTA and DHT treatments.

## 4. Conclusions

In this study, small diameter electrospun collagen tube with random continuous smooth nanofibers was successfully fabricated for tissue engineered tubular graft. Because of the denaturation of collagen caused by dissolving it in conc. aq. acetic acid, electrospun collagen tube lost the nanofibrous structure and showed the poor performance in a wet state. After ammonia treatment, the triple helix structure of collagen can be partially recovered based on the IR data. In further combination with DHT and GTA crosslinking treatments, collagen tubes not only kept the hydrophilic surface, but also showed good mechanical properties, low swelling ratio and a high resistance to dissolution. By comparison, ammonia-GTA treatment gave a higher efficiency of crosslinking, which enhanced the resistance against the damage of water and completely preserved the nanofiber structure in a wet state. In general, crosslinked treatments were beneficial to the improvements of mechanical properties and degradability of electrospun collagen tubes. Further studies will be focused on the biological compatibility of crosslinked electrospun collagen tubes.

## Figures and Tables

**Table 1:** Diameter of collagen nanofibers before and after various treatments.

Treatments	Untreated	Ammonia	Ammonia-DHT	Ammonia-GTA
Fiber diameter/nm	336 ± 149	452 ± 174	439 ± 242	430 ± 227
Increasing ratio/%	-	34.5	30.7	28.0

## Data Availability

The data presented in this study are available on request from the corresponding author.

## Supplementary Materials

The following are available online at https://www.mdpi.com/2073-4360/13/5/755/s1, Figure S1: Tensile strength of collagen tubes with ammonia treatment for various periods of time in a wet state, Figure S2: Tensile strength of collagen tubes with DHT or ammonia-DHT treatment at various temperature for 3 days in a wet state, Figure S3: Tensile strength of collagen tubes with GTA or ammonia-GTA treatment for various periods of time in a wet state.
