# Supplementary material for: Fabrication and Properties of Electrospun Collagen Tubular Scaffold Crosslinked by Physical and Chemical Treatments
Source: Polymers (Basel). 2021 Feb 28;13(5):755. doi: 10.3390/polym13050755 (PMC7957483; doi:10.3390/polym13050755)
Supplement: Supplementary file 1 [file polymers-13-00755-s001.pdf]

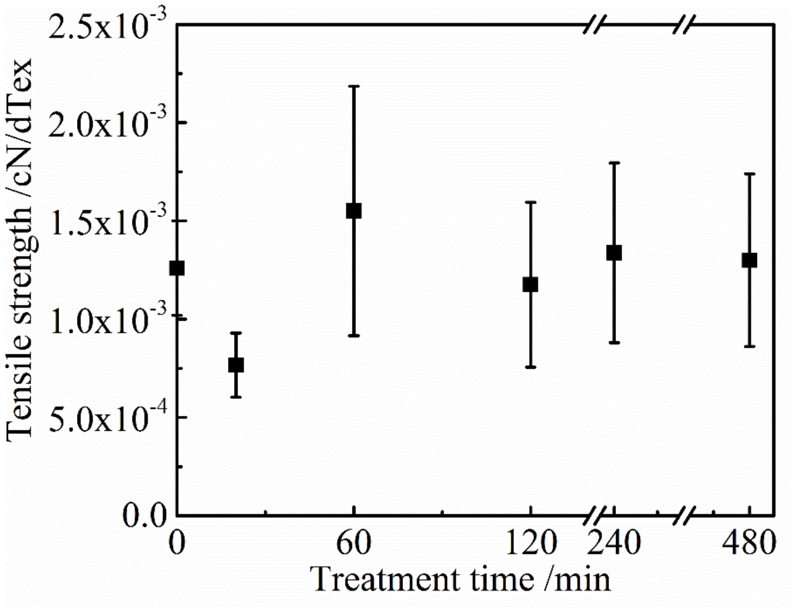

Fig. S1 Tensile strength of collagen tubes with ammonia treatment for various periods of time in a wet state.

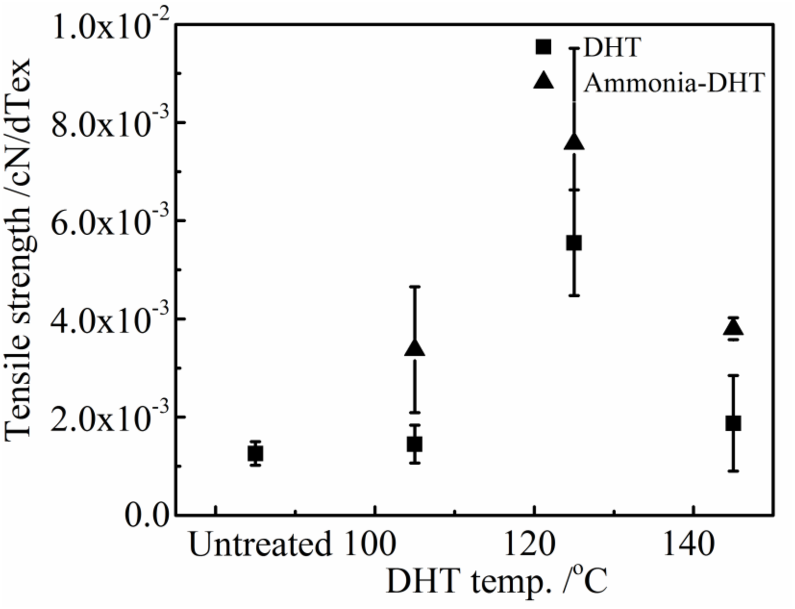

Fig. S2 Tensile strength of collagen tubes with DHT or ammonia-DHT treatment at various temperature for 3 days in a wet state.

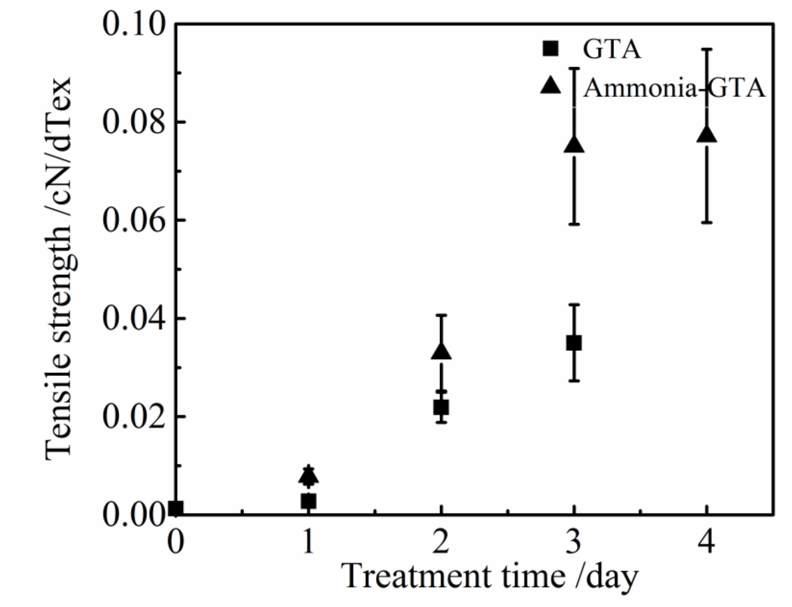

Fig. S3 Tensile strength of collagen tubes with GTA or ammonia-GTA treatment for various periods of time in a wet state.
